# Safety and Effectiveness of an Integrative Treatment of Acupuncture-Based Intervention in Survivors of Breast Cancer With Postmastectomy Pain Syndrome: Protocol for a Single-Center, Single-Arm Exploratory Trial

**DOI:** 10.2196/94381

**Published:** 2026-06-11

**Authors:** Naruaki Kawasaki, Hiroto Ishiki, Naho Matsubara, Takako Ikegami, Ayaka Ishikawa, Sayaka Arakawa, Asuka Takahashi, Tomoko Uchida, Yukie Nameki, Yoko Horiguchi, Takeshi Murata, Shin Takayama, Akihiko Suto, Kota Kihara, Keisuke Ariyoshi, Shunsuke Oyamada, Ayako Kayano, Hiromichi Matsuoka, Tatsuya Takagi, Eriko Satomi

**Affiliations:** 1 Department of Palliative Medicine, National Cancer Center Hospital, 5-1-1, Tsukiji, Chuo-ku, Tokyo, 1040045, Japan, 81 335422511; 2 Department of Palliative Medicine, Graduate School of Medicine, Juntendo University, Bunkyo-ku, Tokyo, Japan; 3 Department of Breast Surgery, National Cancer Center Hospital, Chuo-ku, Tokyo, Japan; 4 Japanese Organisation for Research and Treatment of CancerArakawa-ku, Tokyo, Japan; 5 Department of Psycho-Oncology, National Cancer Center Hospital, Chuo-ku, Tokyo, Japan

**Keywords:** acupuncture, postmastectomy pain syndrome, breast cancer survivorship, chronic pain, study protocol

## Abstract

**Background:**

Postmastectomy pain syndrome (PMPS) is a prevalent chronic pain condition that occurs after breast cancer surgery, often impairing quality of life in survivors of breast cancer. Despite its prevalence, no standardized treatment has been established. Acupuncture has been reported to be an efficacious intervention for the management of chronic pain and may be an effective treatment for PMPS.

**Objective:**

This study aims to explore the effectiveness and safety of integrative treatment of acupuncture-based intervention for PMPS.

**Methods:**

This is a single-center, single-arm, prospective interventional study. Eligible participants are patients with breast cancer who experience chronic postoperative pain in the chest, neck, or shoulder with a numerical rating scale (NRS) score ≥4, are at least 6 months postcurative treatment, have no evidence of disease recurrence, and are not receiving anticancer treatment at enrollment. Participants will receive the intervention once weekly for 12 sessions, followed by a 4-week observation period. The primary end point is the change in the average pain NRS score from baseline to week 16.

**Results:**

The study commenced in October 2023 and is scheduled to continue through March 31, 2027. The first participant was enrolled on October 24, 2023. As of the manuscript submission, 24 patients have been enrolled.

**Conclusions:**

This study will explore the potential role of an integrative treatment of acupuncture-based intervention in the management of PMPS. The results will contribute to the evidence base for acupuncture in PMPS and inform the design of future clinical studies.

## Introduction

Breast cancer is the most common cancer among women and is treated with a multidisciplinary approach, including surgery, radiotherapy, anticancer drugs, and hormonal therapy [1]. The prognosis for early-stage breast cancer is generally favorable, with a 5-year survival rate of more than 85% [2]. Although long-term survival has improved, many survivors experience persistent treatment-related complications, including pain, upper limb dysfunction, lymphedema, and chemotherapy-induced peripheral neuropathy [3-7]. Among these, chronic pain is prevalent and persistent [8-10].

Postmastectomy pain syndrome (PMPS) is a chronic pain condition that develops after breast cancer surgery and is characterized by stabbing, aching, or burning sensations in the chest, neck, or shoulder [11]. It has been reported to affect 20% to 50% of patients following surgery [12-15] and to impair quality of life in survivors of breast cancer [16]. Various treatments for PMPS have been reported, including surgical interventions (such as fat grafting and peripheral nerve surgery), cutaneous laser therapy, nerve blocks, adjuvant analgesics for neuropathic pain, and physical therapy [17-23]. Although the potential benefits of nonpharmacological treatments have been reported, the available evidence remains limited, and no standardized treatment has been established [24].

Acupuncture has been shown to be beneficial for the management of chronic noncancer pain and cancer-related pain [25-28]. The Society for Integrative Oncology and American Society of Clinical Oncology guideline states that acupuncture may be offered for general cancer pain and musculoskeletal pain [29]. The Survivorship Care Guidelines of the American Society of Clinical Oncology Breast Cancer include acupuncture as a recommended treatment option for chronic pain [30]. However, the specific efficacy of acupuncture for PMPS has not been differentiated. Evaluating the effectiveness of integrative treatment of acupuncture-based intervention, as practiced in Japanese clinical settings, may contribute to establishing a novel nonpharmacological treatment option and addressing an unmet clinical need in breast cancer survivorship care.

## Methods

### Study Objective

The objective of this study is to explore the effectiveness and safety of integrative treatment of acupuncture-based intervention for PMPS.

### Study Design and Setting

This is a single-center, single-arm, prospective interventional study. This study will be conducted according to the protocol based on the Standards for Reporting Interventions in Clinical Trials of Acupuncture guideline [31].

### Ethical Considerations

This study protocol was approved by the Institutional Review Board of the National Cancer Center Hospital (research subject number 2023‐091). Written informed consent will be obtained from all participants prior to enrollment. Participants will not receive any financial compensation for participation in this study.

### Eligibility Criteria

The inclusion criteria are as follows: pathologically diagnosed stage I to III breast cancer; ≥6 months since curative surgery with no evidence of recurrence; undergoing observation without anticancer treatment (endocrine and anti–human epidermal growth factor receptor 2 therapies are permitted); pain in the chest, neck, or shoulder on the surgical side that developed after surgery; a pain numerical rating scale (NRS) score ≥4 during the past 24 hours; age ≥18 years; an Eastern Cooperative Oncology Group Performance Status (ECOG PS) of 0 or 1; a neutrophil count ≥1000/μL and a platelet count ≥50,000/μL within 28 days before enrollment; and no change in pain medication within 7 days prior to enrollment.

The exclusion criteria are as follows: upper limb lymphedema on the surgical side; bilateral pain in the chest, neck, or shoulder; painful comorbidities (such as fibromyalgia and arthritis); severe psychiatric disorders; severe chronic comorbidities (such as cardiovascular, hepatic, renal, gastrointestinal, and hematologic diseases); pregnancy or breastfeeding; and a history of previous acupuncture for PMPS.

### Recruitment and Consent

Participants will be recruited from the outpatient setting of the Department of Palliative Care. Patients diagnosed with PMPS and referred to the Department of Palliative Care for pain management will be screened for eligibility. The palliative care physician will provide a detailed explanation of the study and obtain written informed consent. Participation in the study will be entirely voluntary, and patients will be informed that refusal to participate will not affect their clinical care.

### Concomitant Medications

Participants will be required to maintain stable doses of medications that may influence pain throughout the study period. These include opioids (morphine, oxycodone, hydromorphone, fentanyl, tapentadol, tramadol, and methadone), antidepressants, anticonvulsants, benzodiazepines, acetaminophen, nonsteroidal anti-inflammatory drugs, and corticosteroids.

Initiation of new analgesic medications during the study period will not be permitted. Any unavoidable changes in medication will be documented. However, as-needed benzodiazepine use for sleep will be permitted.

### Intervention

The intervention will be administered by licensed acupuncturists with at least 5 years of clinical experience. All acupuncturists will complete a standardized training program for the study intervention (3 days, 8 hours/day; total 24 hours) prior to study initiation.

The intervention consists of acupuncture, moxibustion, hot pack therapy, and self-care instructions. Acupuncture is the primary treatment, while moxibustion, hot packs, and self-care instructions are considered supplementary.

Acupuncture is performed in the Japanese style and includes static needling, motion needling, and teishin (noninsertive acupuncture). Disposable stainless steel needles (0.16‐0.20 mm in diameter and 40‐50 mm in length; SEIRIN) are used for static and motion needling. For static needling, needles are inserted to a depth of approximately 5 mm and retained for 5 to 10 minutes. For motion needling, needles are inserted to a depth of 5 to 20 mm. After needle insertion, slow passive movements are performed 2 to 3 times, including wrist flexion or extension; forearm pronation or supination; upper arm rotation; scapular protraction or retraction; and shoulder flexion, extension, and abduction. Elicitation of a muscle twitch response is not required.

Teishin is performed using nonpenetrating metallic instruments (approximately 1.0‐12.0 mm in diameter and 110 mm in length), applying gentle compressive stimulation with light rubbing for 1‐ to 2 minutes.

**Figure 1.**
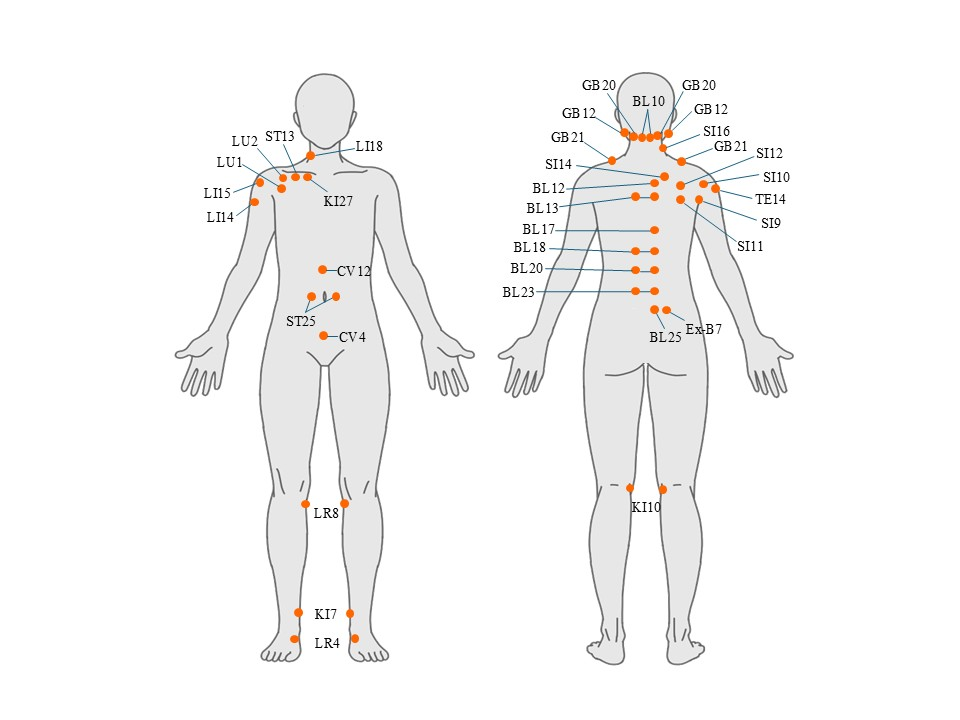
Essential acupuncture points (for right-sided postmastectomy pain syndrome).

Acupuncture points are listed in Tables1  2. Essential points (Figure 1) are applied to all participants, and additional points are selected according to individual symptoms and findings on physical examination. If shoulder range of motion is restricted, additional needles will be applied to the affected muscles.

**Table 1.** Essential acupuncture points.

		Essential points
Unilateral		
	Static needling	BL25, CV4, CV12, LI18, SI16, and Ex-B7
	Static needling and teishin	LU1, LU2, ST13, and KI27
	Static needling and motion needling	LI14, LI15, SI9, SI10, SI11, SI12, SI14, TE14, BL12, and BL17
Bilateral		
	Static needling	BL10, BL13, BL18, BL20, BL23, GB12, GB20, GB21, KI7, KI10, LR4, LR8, and ST25

**Table 2.** Additional acupuncture points.

		Additional points
Unilateral		
	Static needling and motion needling	LU6, LI10, PC4, and TE9
	Static needling, motion needling, and teishin	Pectoralis major muscle, supraspinatus muscle, infraspinatus muscle, subscapularis muscle, rhomboid muscle, deltoid muscle, biceps brachii muscle, triceps brachii muscle, trapezius muscle, latissimus dorsi muscle, teres major muscle, teres minor muscle, and levator scapulae muscle
Bilateral		
	Static needling	ST36, BL56, BL57, and BL60

Moxibustion and hot pack therapy are applied to areas of the chest wall, axilla, and upper limbs presenting abnormal sensations. After each treatment session, participants receive self-care instructions, including self-massage of the anterior chest wall, axilla, and upper limb, as well as stretching exercises to improve shoulder range of motion.

### End Point

The primary end point is the change in the average pain NRS score from baseline to week 16.

Secondary end points include the following:

- Changes in average pain NRS score, shoulder joint range of motion, Shoulder36, European Organisation for Research and Treatment of Cancer Quality of Life Questionnaire Core 30 (EORTC QLQ-C30), and the five viscera score assessed every 4 weeks from baseline
- Changes in Hospital Anxiety and Depression Scale (HADS) and Edmonton Symptom Assessment System (ESAS) scores from baseline to week 16
- The proportion of participants achieving 1-, 2-, and >2-point reductions in pain NRS score at week 16 compared with baseline
- The proportion of participants achieving ≥30% and ≥50% reductions in pain NRS score at week 16
- Time to achieve a 2-point reduction in average pain NRS score
- The proportion of participants achieving their personal pain goal at week 16
- Neutrophil and platelet counts at week 12

Adverse events will be evaluated according to the Common Terminology Criteria for Adverse Events, version 5.0.

### Study Schedule

**Figure 2.**
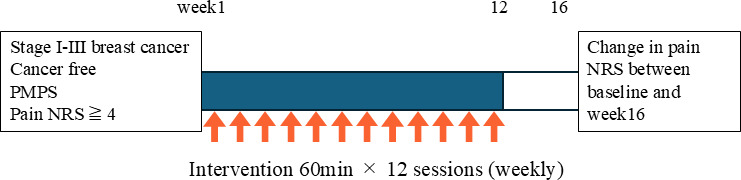
Trial procedure flow. NRS: numerical rating scale; PMPS: postmastectomy pain syndrome.

The study schedule is presented in Table 3. The intervention will commence within 28 days of enrollment and will be administered once weekly for 12 sessions. A 4-week follow-up period will be conducted after completion of the intervention phase (Figure 2).

**Table 3.** Study schedule.

	Enrollment	Weeks 1‐12	Weeks 4, 8, and 12	Week 12	Week 16
Informed consent	✓				
Laboratory data	✓			✓	
Eligibility screening	✓				
Personal pain goal	✓				
ECOG PS^a^	✓		✓		✓
Intervention		✓			
Pain NRS^b^	✓	✓			✓
Shoulder joint range of motion	✓		✓		✓
Shoulder36	✓		✓		✓
EORTC QLQ-C30^c^	✓		✓		✓
Five viscera score	✓		✓		✓
ESAS^d^	✓				✓
HADS^e^	✓				✓
Adverse event			✓		✓

a ECOG PS: Eastern Cooperative Oncology Group Performance Status.

b NRS: numerical rating scale.

c EORTC QLQ-C30: European Organisation for Research and Treatment of Cancer Quality of Life Questionnaire Core 30.

d ESAS: Edmonton Symptom Assessment System.

e HADS: Hospital Anxiety and Depression Scale.

At enrollment, baseline data will be collected, including age, sex, surgical side, treatment history, pain location, and personal pain goal. Blood tests will be performed within 28 days prior to enrollment and at week 12.

Clinical assessments, including ECOG PS and shoulder joint range of motion, will be performed at enrollment and every 4 weeks thereafter. Adverse events will be evaluated every 4 weeks from intervention initiation. Patient-reported outcomes will be collected at predefined time points, as specified in Table 3.

### Discontinuation Criteria and Safety Monitoring

The protocol treatment will be discontinued if any of the following conditions are met: participant request for discontinuation, recurrence of breast cancer, development of serious comorbidities, or adverse events deemed by the physician to preclude continuation of the study intervention.

Safety monitoring will be conducted through assessments by acupuncturists at each intervention session, physician assessments every 4 weeks, and additional physician assessments at the participant’s request. If adverse events are identified by acupuncturists during each session, they will be reported to a palliative care physician, who will provide appropriate evaluation and management.

### Data Collection Methods

Patient-reported outcomes, including pain NRS, Shoulder36, EORTC QLQ-C30, five viscera score, ESAS, and HADS, will be completed by participants using self-administered questionnaires. Clinical assessments, including ECOG PS, shoulder joint range of motion, and adverse events, will be conducted by physicians during scheduled study visits. All study data will be entered into a secure electronic data capture system by trained study personnel.

### Data Management and Monitoring

All data will be collected by the Japanese Organisation for Research and Treatment of Cancer Data Center. Data will be retained in accordance with the Japanese Clinical Research Act. Clinical data management and central monitoring will be conducted using the electronic data capture system VIEDOC 4 (Viedoc Technologies AB). The research data and records will be stored for 10 years after publication of the paper and will then be discarded.

### Sample Size and Statistical Analysis

In this study, we considered that a difference of approximately 2 points in pain scores before and after the intervention, corresponding to the minimal clinically important difference for pain advocated by the Initiative on Methods, Measurement, and Pain Assessment in Clinical Trials, would be clinically meaningful. Therefore, we assumed a mean difference of 2 points in pain scores from baseline to week 16, a SD of 4 points, and a correlation coefficient of 0.5 between preintervention and postintervention values. As a result, the required sample size was calculated to be 27 at a 1-sided significance level of 5% and 80% power. The sample size was set at 30 cases to allow for dropouts during the trial.

The analysis population will include patients who have received at least one dose of the intervention. All data collected during the observation period after discontinuation of the intervention will be included in the analysis. In principle, missing data will not be imputed in this study, and the analysis will be conducted using only the observed values (observed case analysis). However, if missingness is largely attributable to worsening clinical condition, the results of the observed case analysis may be biased toward overestimation. Therefore, sensitivity analyses using appropriate imputation methods will be conducted to assess the robustness of the findings (prepared before data are fixed).

As an analysis of the primary end point, a paired *t* test (1-sided test) will be conducted on the null hypothesis that “the mean difference between the average pain NRS before intervention and at 16 weeks is greater than or equal to zero.” The significance level for this hypothesis is set at 5% for 1-sided testing, and the corresponding 2-sided 90% CI will be calculated for the mean change from baseline to week 16. If the hypothesis testing is significant, it will suggest the effectiveness of the intervention in this exploratory study and support the planning of a confirmatory trial.

For the secondary end points of pain, shoulder joint range of motion, Shoulder36, and HADS, a paired *t* test (2-tailed test) will be conducted using a two-sided significance level of 5% to test the null hypothesis that “the mean value of the change before and after intervention is zero,” and the point estimate and 95% CI of the mean at each time point, as well as for the change from baseline, will be calculated. We will also perform visual assessments based on calculating summary statistics for each time point, plotting the mean changes in the mean with 95% CI, and creating a plot for each patient at each time point. Given the limited sample size, these secondary end points will be treated as exploratory. Changes in pain NRS will be explored in relation to changes in secondary end points, including EORTC QLQ-C30, the five viscera score, the pain target achievement rate, and the ESAS. It is hypothesized that greater improvement in pain will be associated with greater improvement in these end points, indicating a positive association. As this analysis is exploratory, no statistical hypothesis testing will be performed. Instead, the relationships will be assessed using descriptive statistics and visual inspection of scatter plots. For adverse events, the incidence rate for each event will be calculated overall, by grade, and for grade 3 or higher events.

Statistical analyses will be performed using SAS software (version 9.4; SAS Institute).

### Protocol Version

This manuscript is based on protocol version 1.7 (dated January 9, 2025).

## Results

The study commenced in October 2023 and is scheduled to continue through March 31, 2027. The first participant was enrolled on October 24, 2023. As of manuscript submission, 24 patients have been enrolled.

## Discussion

### Principal Results

This study protocol outlines a trial designed to explore the effectiveness and safety of an integrative treatment of acupuncture-based intervention in patients with PMPS. Chronic pain in survivors of cancer is often difficult to manage over the long term, highlighting the need for treatment modalities that do not rely solely on pharmacological interventions. As a nonpharmacological therapy, acupuncture may represent a potential treatment option for PMPS, provided that its safety and potential effectiveness are demonstrated.

A notable strength of this study is that the intervention includes moxibustion, hot pack therapy, and self-care instructions in addition to acupuncture. Previous studies assessing the effects of acupuncture for chronic pain have predominantly evaluated acupuncture alone. The integrative approach adopted in this study may enhance therapeutic effects and offer additional insight into the management of chronic pain, including PMPS. In addition, this trial will assess shoulder function and overall quality of life as secondary end points, enabling a comprehensive evaluation of treatment effects beyond pain reduction to include functional improvement and quality of life.

### Limitations

This study has several limitations. First, the single-arm design without a control group precludes causal inference, making it difficult to distinguish the observed improvements from placebo effects or natural recovery. This design was chosen because this study is intended as an exploratory, hypothesis-generating investigation to assess the effectiveness and safety of an integrative intervention in a real-world clinical setting. Second, the intervention includes multiple components, such as acupuncture, moxibustion, hot pack therapy, and self-care instructions. As a result, the individual contribution of each component cannot be isolated. However, this integrative approach reflects Japanese-style clinical practice, where these therapies are often delivered in combination, and is therefore considered appropriate to evaluate the overall effectiveness in a pragmatic context. Accordingly, the findings should be interpreted as reflecting the effectiveness of the combined intervention rather than acupuncture alone. Third, the small sample size limits statistical power and does not allow for a definitive evaluation of effectiveness. Nevertheless, this study will provide preliminary data that may inform the design and sample size estimation of future trials. Fourth, missing data will not be imputed, and analyses will be conducted based on observed cases only. This approach may introduce bias if the missing data are not completely at random. Finally, this is a single-center study, which may limit generalizability. Future multicenter randomized controlled trials are warranted to evaluate the efficacy of acupuncture-based interventions for PMPS.

### Conclusion

This study will explore the potential role of integrative treatment of acupuncture-based interventions in the management of PMPS. The results are expected to contribute to the evidence base for acupuncture in PMPS and inform the design of future clinical studies.

## Abbreviations

ECOG PS: Eastern Cooperative Oncology Group Performance Status
EORTC QLQ-C30: European Organisation for Research and Treatment of Cancer Quality of Life Questionnaire Core 30
ESAS: Edmonton Symptom Assessment System
HADS: Hospital Anxiety and Depression Scale
NRS: numerical rating scale
PMPS: postmastectomy pain syndrome
